# Systematic Review and Psychometric Properties Analysis of First-, Middle-, and Top-Level Nurse Manager's Core Competencies Instruments

**DOI:** 10.1155/2024/2655382

**Published:** 2024-04-27

**Authors:** Lucia Filomeno, Daniela Forte, Emanuele Di Simone, Marco Di Muzio, Daniela Tartaglini, Marzia Lommi, Dhurata Ivziku

**Affiliations:** ^1^Department of Biomedicine and Prevention, Tor Vergata University of Rome, Via Cracovia, 50 00133 Rome, Italy; ^2^Department of Clinical and Molecular Medicine, Sapienza University of Rome, Via Giorgio Nicola Papanicolau, 00189 Roma RM, Italy; ^3^Head of Healthcare Professions Department, Fondazione Policlinico Universitario Campus Bio-Medico, Via Alvaro del Portillo, 200 00128 Rome, Italy; ^4^Healthcare Professions Department, Fondazione Policlinico Universitario Campus Bio-Medico, Via Alvaro del Portillo, 200, 00128 Rome, Italy

## Abstract

**Purpose:**

Healthcare organisations need to define the role of the nurse manager in light of recent global health developments. For this purpose, several core competencies essential for each hierarchical management level need to be assessed. Different measurement instruments have been developed to assess nurse managers' competencies. This systematic review summarises the characteristics and psychometric properties of existing instruments measuring first-, middle-, and top-level nurse managers' competencies.

**Methods:**

Following PRISMA guidelines for reporting and COnsensus-based Standards for the selection of health Measurement INstruments (COSMIN) guidelines, 789 articles were retrieved from PubMed, Scopus, CINAHL, and APA PsycINFO databases with no time limitation. The review protocol was registered on PROSPERO (CRD42023425854).

**Results:**

Ten tools were identified, assessing one or more competencies among nurse managers: Competency Elements for Nurse Managers of Tertiary General Hospitals, NICA-NL, HCCI, I-FLNMMCS, NMCI, Chase Nurse Manager Competency Questionnaire, CASHN, Questionnaire for Head Nurses' Managerial Competencies, Nurse Manager EBP Competency Scale, and the Home Healthcare Nurse Manager Assessment Tool.

**Conclusion:**

Following the COSMIN assessment, the Chase Nurse Manager Competency Instrument was the most comprehensive among the included instruments, and the CASHN questionnaire scored highest on methodological quality and level of evidence. These instruments can be used in clinical practice to evaluate competencies and as a basis for developing managerial training courses.

## 1. Introduction

The literature extensively discusses the concept of competence, but notable discrepancies in its definition hinder consensus. This lack of clarity often arises from using specific frameworks across diverse contexts and disciplines, revealing significant disparities in the interpretation and application of key terms related to competence and understanding of their interrelation. European Directive 2006/962/EC suggested exploring competencies linked to specific disciplines for a better understanding. One of the earliest and widely used definitions by Hamel and Prahalad [1] characterizes competence as the intersection of knowledge, skills, attitudes, and values, mobilized to address diverse situations. This perspective recognizes the intricate interplay between competence and other aspects that contribute to performance. Clinical psychologist Robert White defines competence as a fundamental human effort motivating individuals, asserting that competence and activities fostering it lead to inspirational pathways to success [2].

Several interpretative approaches view competence as a function of the context in which it is applied, where “worker and work form one entity through the lived experience of work” [3]. Within this framework, competencies go beyond learned contents/concepts, imposing a profound reorganisation of acquired knowledge and relative transfer in the labour market. Moreover, it is essential to consider that discipline-specific competencies represented only 30% of the entire cluster of competencies, while the remaining 70% were expected to have a wide range of specialisations [4].

In healthcare management, certain competencies, such as planning and managing resources or supporting teamwork and communication, are common to other specialisations. In contrast, nursing-specific competencies have become the subject of study in more recent research over the last ten years and were approached from various perspectives. For example, Chase [5] identified competencies such as technical, human, conceptual, and leadership and organised quality care services. On the other hand, the American Organization of Nurse Leaders has identified competencies such as the ability to create and maintain good relationships, communication, leadership, knowledge of the healthcare environment and clinical principles, professionalism, business skills, and strategic leadership as key areas in developing competencies [6, 7].

Effective management competencies constitute the cornerstone of success and efficiency in healthcare organisations. Competent nurse management fosters the quality of patient care and contributes to favourable employee morale and engagement, reducing turnover rates and cultivating a positive workplace culture [8, 9]. According to a systematic review, competencies such as planning, communication, and leadership are associated with enhanced patient satisfaction, increased nurses' job satisfaction, and reduced occurrences of patient mortality, prescription mistakes, restraint usage, and hospital-acquired infections [8].

Different levels of healthcare organisational management and governance require distinct competencies for effective role application in this scenario. Top-level nurse managers (NMs) engage in higher-level planning, such as mission and strategy, which require more excellent conceptual skills [10]. Middle-level NMs deal with specific processes to deliver value, necessitating technical skills to manage their area of specialisation [11, 12].

First-level NMs are leaders of units in hospitals or other medical settings. Their role does not include regular interaction with patients. Instead, they influence the quality of healthcare by leading the work and helping to ensure that the medical facility operates smoothly [13, 14].

Competence-related conceptual models combine skills, abilities, and knowledge encompassing specific behaviours that an individual exhibits. Competencies are typically categorised into three main types, as described by Robert L. Katz, who developed a framework to explain managerial competencies [15]. In his 3 Skills Taxonomy, he set out the following categories:

- Conceptual, as the formulation of ideas. Managers understand the importance of relationships, developing ideas, and problem-solving.
- Human involves the ability to interact with people. Managers interact and cooperate with employees, patients, and superiors.
- Technical involves knowledge and proficiency in managing processes. Managers use specific tools for one particular area.

Dreyfus [16] identified five stages of progress in competencies: novice, advanced beginner, competent, proficient, and expert. Their model assumes that the longer one practices, the more competent one becomes at a job or task. Acquisition of competencies is a matter of experience; each stage requires time and practice.

Regarding more nursing-specific concept models, a scoping review conducted by González-García et al. [17] identified 22 competencies grouped into six dimensions: management, communication and technology, leadership and teamwork, knowledge of the health system, nursing knowledge, and personality. Based on that, the same authors created a nurse executive competency model (MCGE-executive level) to define the executive nurse's position, expected level of performance, and development required. It is composed of 51 competencies, tracking progress in each from “competent” to “expert” [10].

The current research in nursing and management endeavours to delineate essential competencies required for nurse managers, stratifying them based on the level of management. The absence of a comprehensive description of competencies and how they manifest at different managerial levels poses a challenge in effectively measuring the enrolment and evaluation of nurse managers. There is a pressing need for an instrument capable of measuring nurse manager competencies across various hierarchical levels, facilitating our understanding of the nuanced application of these competencies in real-world healthcare settings. In order to identify appropriate instruments to measure NMs' competencies, this study summarises existing instruments for assessing and evaluating core competencies of first-, middle-, and top-level nurse managers.

## 2. Methods

A systematic review was carried out to examine existing instruments assessing the core competencies of nurse managers according to COnsensus-based Standards for the selection of health Measurement INstruments (COSMIN) guidelines [18]. The Preferred Reporting Items for Systematic Reviews and Meta-Analysis (PRISMA) checklist was used to describe the study selection process [19]. The COSMIN guidelines were used for the quality assessment of the included articles.

The review protocol was registered on PROSPERO with ID record no. CRD42023425854.

### 2.1. Search Strategy

A search was performed on the following four online bibliographic databases: PubMed, CINAHL, Scopus, and APA PsycINFO. Search terms were based on a COSMIN search filter to identify studies of psychometric properties, combined with terms relevant to nurse manager competencies.  Table 1 summarises the search strategy used and the main terms included. We also hand-searched the reference lists of articles identified for inclusion in the review to uncover additional relevant studies.

To guide the synthesis of selected articles, Robert L. Katz's framework [15] was used as a methodological approach to organise the results and discover patterns and commonalities. The research team chose this specific framework because, to date, it is the most referred to from studies conducted in this area of interest.

The selection of studies was carried out from database inception to March 30th, 2023.

The PICO (population, instruments, construct, and outcomes) was formulated as follows:

- P: nurse managers at every hierarchical level
- I: instruments assessing core competencies through validation studies and psychometric measurements testing
- C: instruments' constructing the building process
- O: to establish a level of recommendation based on measurement properties

### 2.2. Inclusion and Exclusion Criteria

The inclusion criteria were as follows: (i) development and validation studies of instruments assessing NMs competencies (at every level: first, middle, and top management and in different settings); (ii) grey literature such as dissertations (only doctoral); (iii) published peer-reviewed studies; and (iv) articles written in English and Italian. No time limitation was applied.

The exclusion criteria were as follows: (i) qualitative and quantitative studies that did not have as their main goal the development, psychometric testing, and validation of a new scale for managerial competencies (e.g., surveys, cross-sectional or phenomenological design, protocols, or reviews); (ii) studies that tested the developed instrument on core competencies of managers in nursing samples; (iii) studies that did not publish the instrument in the paper; or whose instruments were not in English. The researchers contacted the authors of such scales to ask for the instrument and/or its availability in English. The study was excluded if no answers were received or if the instrument was not available in English.

To provide a comprehensive and up-to-date overview, we decided to report in this study only the most recent version or modification of an instrument that has been validated. However, when evaluating quality, we also considered previous validation publications. Of these, cross-cultural validation studies and those that presented limited and/or insufficient validation data (e.g., only Cronbach's alpha and no exploratory factor analysis) were excluded.

### 2.3. Data Extraction and Synthesis

Two researchers (LF and DI) independently screened the titles and abstracts of the articles identified by the search strategy. Disagreements about inclusion or exclusion were resolved by consulting a third researcher (EDS), who is a supervisor or senior member of the team who has major knowledge and experience on the topic. Articles that potentially met our inclusion criteria but whose supporting information was insufficient for inclusion were retrieved.

In the final phase, interresearcher agreement on inclusion and exclusion was calculated as Cohen's kappa. The scores of the two independent researchers were compiled and compared. The agreement coefficient was *κ* = 0.67 (86.7%), indicating a substantial agreement [20].

In accordance with the COSMIN manual for systematic reviews of PROMs [21], data were synthesised reporting the following:

- The characteristics of the included PROMs, such as the name of the PROMs, reference to the article in which the development of the PROM is described, constructs being measured, language and study population for which the PROM was developed, the intended context of use, the available language version of the PROM, number of scales or subscales, number of items, response options, recall period, interpretability aspects, and feasibility aspects;
- The characteristics of the included study population;
- The methodological quality ratings of each study per measurement property;
- A Summary of Findings' (SoF) table per measurement property.

### 2.4. Quality Appraisal

Three independent researchers (LF, DI, EDS, and ML) assessed included studies' methodological quality and psychometric properties under the COnsensus-based Standards for the Selection of Health Measurement INstruments (COSMIN) checklists [21].

First, the researchers evaluated each instrument development study and content validity against ten quality criteria defined in the COSMIN checklist. An overall rating for instrument development and content validity was determined based on the quality and results of the available studies. For each instrument, the researchers summarised the results as sufficient (+), insufficient (−), inconsistent (±), or indeterminate (?) and classified the quality of the evidence as high, moderate, low, or very low, using a modified GRADE approach based on the risk of bias (quality of the studies), inconsistency (of the results of the studies), and indirectness (evidence comes from different populations, interventions, or outcomes from those of interest in the review) [18].

Second, researchers evaluated the instruments' construct validity, reliability, and responsiveness. The COSMIN checklist assesses whether a study meets the standards for good methodological quality on a four-point rating scale (very good, adequate, doubtful, inadequate), classifying the psychometric properties as sufficient (+), insufficient (−), or indeterminate (?) and assigning a quality of evidence as high, moderate, low, and very low.

Finally, an overall recommendation is made using the grading of recommendations assessment, development, and evaluation (GRADE) approach, assigning level A of recommendation for use in higher-quality studies, level B for studies potentially recommended but in need of further testing, and level C for studies not recommended for use. Level B is assigned when the scale cannot be classified as level A or C.

## 3. Results and Discussions

Overall, 789 publications were retrieved from the electronic databases. After removing duplicates and studies that did not fulfil the inclusion criteria, the authors were left with 10 studies included in the review that were eligible for methodological quality assessment.

**Figure 1:**
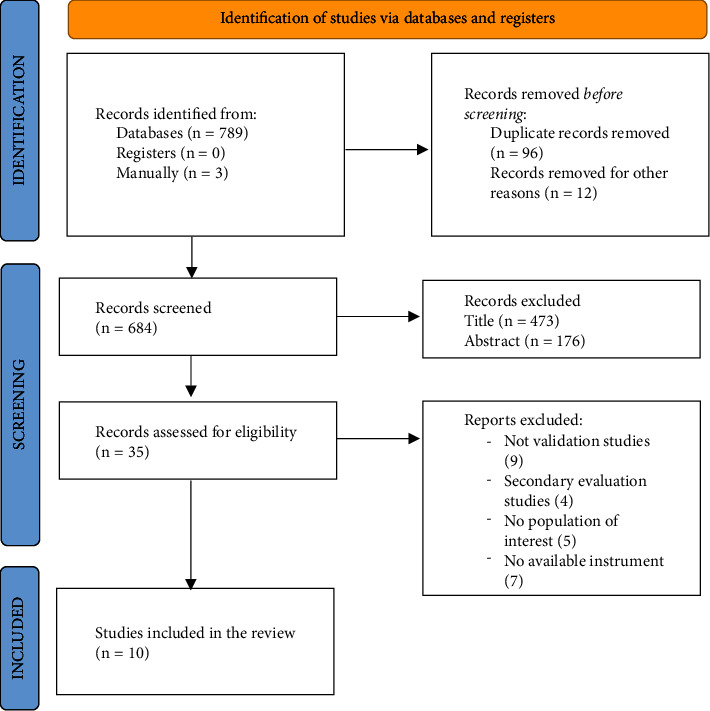
PRISMA 2020 flow diagram [19].

The PRISMA flow chart of study selection is presented in Figure 1.

### 3.1. Characteristics of Included Studies and Instruments

Overall, 10 studies were included in the review. The studies were conducted in different countries: USA (6), Iran (1), China (1), Thailand (1), and Indonesia (1). A detailed information on studies is summarised in Table 2.

The studies targeted competencies of different nurse managerial categories such as *supervisors*, *directors*, *head nurses*, *chief nurses,* and others. In some studies, a clear division of managers into first-level, middle-level, and top-level categories was presented, specifying the sample size for each category [22–26], whereas in other studies, the sample was described in an aggregated form [5, 27–29].

All reviewed studies were conducted in main hospital settings except for two that were conducted in a tertiary general hospital [22] and in a home healthcare nursing agency [26]. The instruments were self-reporting with structured response options of 3- to 5-point Likert scales. The number of items ranged from 16 [25] to 93 items [29], and the instruments were tested on samples of managers ranging from 30 to 614 persons.

### 3.2. Psychometric Properties and Methodological Quality of Instruments

The quality of evidence and the psychometric properties of the development and validation studies of the instruments are presented in Table 3.

Overall, one study was of high quality [28], 8 instruments [5, 22, 23, 25–30] presented moderate quality of evidence in the content validity, and one was of low quality [24].

The most common areas of bias were instrument development procedures (doubtful qualitative methodology for finding relevant items; doubt over the presence of any trained moderator/interviewer; lack of interview guidance in the article; a doubtful process of recording/transcribing participant's responses; doubt over the independence of the data decoding process; and doubt whether data saturation was reached).

In pilot tests, bias was assigned to doubtful relevance, completeness, or clarity of items to the respondent and to the low numbers of participants enrolled in the pilot test/expert panel.

Regarding the construct's psychometric properties, one study [28] presented high quality on internal consistency and structural validity, 4 studies [5, 22, 29, 30] presented moderate quality, 4 studies [24–27] were of low quality, and 1 study [26] was of very low quality. Low scores for structural validity were given when the sample size used in the analysis was inadequate (adequate rating = at least 5x and ≥100, or 6x and <100).

Overall, five instruments were given a GRADE A rating [25, 27–30], and five instruments a GRADE B rating [5, 22–24, 26] because, in most of them, the sample size did not satisfy the requirement of at least 5 times the number of items and ≥100 or at least 6 times the number of items but <100.

### 3.3. Instruments Descriptions According to Quality Level and Managerial Domains Explored

The instruments included in this review varied in the complexity of the competencies explored. Some scales included 17 domains of competencies, whereas others included only 4. The details are presented in Table 4. Four scales [5, 27, 28, 30] explored the greatest number of competencies.

Some competencies were commonly included in more than 6 instruments, including staff advocacy and development, team communication and collaboration, time management, quality improvement, leadership, problem-solving, and evidence-based practice. However, other competencies, such as organisational and policy overview or negotiation, were mentioned only by single instruments [5, 27].

- Nurse Manager Competency Inventory (NMCI).
- The NMCI measures competencies in building cohesive teams by fostering a collaborative and supportive environment [25]. This scale explored the following job competency domains: staff retention, staff recruitment, staff development, performing supervisory responsibilities, quality care, conducting daily unit operations, fiscal planning, communication, quality improvement, promoting a professional practice model, and developing self.
- Cronbach's *α* was calculated and reported only for item-total correlations, giving a “moderate” quality of evidence (QoE) in content validity and “low” QoE in structural and construct validity and internal consistency. Only this study measured responsiveness data before and after intervention with effect size. The respondent's role by itself accounted for only 2-3% of the total overall variance. The measurement properties quality (MPQ) was “sufficient.” This resulted in a GRADE A level of recommendation.
- The Human Capital Competencies Inventory (HCCI) for nurse managers.
- The HCCI is a self-assessment instrument for measuring NMs' competencies in managing human capital [24]. Content validity testing (*N* = 3 and CVI = 1.0, for all retained items) and internal consistency reliability (*N* = 99; Cronbach's alpha = 0.84–0.89) yielded 58 activities in five subscales: developing self, recruiting, developing others, utilising, and retaining.
- This development study was not performed in a sample representing the target population, resulting in a “low” QoE in every psychometric property measured. Moreover, reviewers evaluated how the items were worded and whether the response options matched the question as “undetermined.” Therefore, the MPQ for content validity was “insufficient,” as was the structural validity. Internal consistency and construct validity were rated as “sufficient” because Cronbach's alpha was calculated for each unidimensional subscale separately, and an adequate description of the important characteristics of the subgroups was provided. The inventory was finally given a GRADE B.
- Chase Nurse Manager Competency Instrument.
- The authors used the five domains of Katz's conceptual framework (technical, human, conceptual, financial, and leadership), confirmed by a PCA.
- Test-retest reliability was conducted by administering the same test twice, at a two-week interval, to a group of 23 NMs (*r* = 0.88). With regard to comparing tools, Chase [5] was the only researcher who could compare his latest scale version with the first one developed in 1994.
- The Chase Nurse Manager Competency Instrument was the only one validated in other languages (Hebrew and Slovenian) by other authors [31, 32]. Experts produced the translations independently and performed multiple forward and backward translations. Still, no numerous group factor analysis or differential item functioning (DIF) analysis was reported as COSMIN guidelines recommend.
- These results allowed the reviewers to rate all properties as “moderate.” A “sufficient” QoE was given to all measurements except for structural validity (inadequate sample size). The instrument was finally assigned a GRADE B.
- Home Healthcare Nurse Manager Assessment Tool.
- The tool developed by Rosenfeld et al. [26] focused on home healthcare nurse managers' competencies, comprised of five domains: leadership, problem-solving, planning and organisation, coaching, and aligning performance for success, tested with a PCA with varimax rotation explaining 66.9% of the variance. Cronbach's alpha for each subscale ranged from 0.866 to 0.948 and weighted kappa from 0.58 to 0.86.
- The tool scored “moderate” QoE except for structural validity, internal consistency, and reliability, where it scored “very low.” Reviewers gave these scores because of inadequate sample size, the absence of any clear description of the construct, and the lack of a qualitative method to assess comprehensibility. This tool was allotted a GRADE B recommendation.
- Competency Assessment Scale for Head Nurses (CASHN).
- The CASHN tool was developed by Tongmuangtunyatep et al. [28] to evaluate head nurses' competencies in community hospitals. The final version comprised five factors: leadership, healthcare environment management, policy implementation and communication, management, and professional ethics.
- The CASHN presented an I-CVI ranging from 0.83 to 1.00, and the S-CVI was 0.94. The internal consistency reliability ranged from 0.93 to 0.96.
- This scale was the only one that scored “high” and “sufficient” in every measurement property, reaching a GRADE A. Therefore, we highly recommend this instrument for future cultural validation studies.
- Nursing Informatics Competency Assessment for the Nurse Leader (NICA-NL).
- The NICA-NL instrument was specifically developed to assess a set of informatics competencies relevant to NMs [26].
- The scale comprised 26 items and six domains: strategic implementation, advanced information and education, executive planning, ethical and legal concepts, information systems concepts, and requirements and system selection.
- Cronbach's alpha ranged from 0.81 to 0.96.
- This scale scored “low” for structural validity and internal consistency. Only EFA was performed, and the sample size included in the analysis was inadequate. Neither qualitative nor quantitative methods to assess comprehensibility were described. The final GRADE was B.
- Nurse Manager EBP Competency Scale.
- Shuman et al. [29] developed an entire instrument focused on measuring NMs competencies regarding EBP consisting of 16 items and two domains: EBP knowledge and EBP activity. The subscales demonstrated reliabilities of 0.90 (95% CI = 0.87 and 0.93) and 0.94 (95% CI = 0.92 and 0.96), respectively. Cronbach's alpha for the entire scale was 0.95.
- This scale was rated “moderate” and “sufficient” in all the psychometric properties except for “indeterminate” in structural validity due to inconsistent sample size. The scale has a GRADE A of recommendation.
- Questionnaire for Head Nurses' Managerial Competencies.
- Moghaddam et al. [27] initially developed a competency model to provide a valid tool for assessing the managerial competencies of hospital department head nurses. This tool measured 27 competencies categorised by four main managerial tasks: planning, organising, leadership, and control (Cronbach's alpha = 0.93; ICC = 0.89).
- Results revealed that the study population gave the highest priority to strategic thinking (0.122) and the lowest to evidence-based decision-making (0.007).
- The questionnaire scored “sufficient” for the methodological quality of each assessed property. Internal consistency and reliability were rated as “low” because statistics were not calculated for each unidimensional subscale separately, and the time interval (recall period) for face validity was not clearly stated. The scale reached a GRADE A level of recommendation.
- Indonesian First-Line Nurse Managers' Managerial Competence Scale-I (FLNMMCS).
- This study developed a practical, 43-item instrument (I-FLNMMCS) with 7 domains: leadership, facilitating spiritual nursing care, self-management, staffing and professional development, informatics, financial management, and applying quality care improvement to evaluate the managerial competence of Indonesian FLNMs (Cronbach's alpha = 0.955; CVI = 0.859; *r* = 0.321–0.687) [30].
- The scale underwent a forward-backward translation method from Indonesian to English (CVI from 0.83 to 1).
- The scale achieved “moderate” QoE and adequate methodological quality for the measured properties, resulting in GRADE A.
- Competency Elements for Nurse Managers of Tertiary General Hospitals.
- This study examined the NM competency model of tertiary general hospitals in China [22]. The instrument consists of 22 competencies and four dimensions: leadership and management ability, personal traits, professional quality, and professional ability, and includes elements of proper staffing/scheduling, hiring/recruiting, developing staff competencies, role-modelling, retaining staff, and coaching/mentoring professionals (Cronbach's alpha = 0.745–0.885; KMO = 0.928).

The reviewers rated all the psychometric properties as “moderate,” with some differences for the QoE. Relevance and comprehensiveness were rated as “insufficient” because the origin of the construct was not clear (a theory, conceptual framework, disease model, or clear rationale provided to define the construct to be measured), and it was doubtful whether skilled interviewers were used for concept elicitation. Structural validity was also “insufficient” because only EFA was performed. The final GRADE was B.

### 3.4. Discussions

This systematic review aimed to summarise the characteristics and psychometric properties of existing instruments measuring first-, middle-, and top-level nurse managers' competencies. The review evidenced that some instruments explored a broad range of competencies, addressing core competencies and major themes such as change and resource issues, leadership and management, teamwork and communication, finance, informatics, and technology. Other instruments explored at a deeper level single competencies such as the EBP competency scale [29] and the NICA-NL tool for informatics [23]. Furthermore, among the included instruments, only the Chase Nurse Manager Competency Instrument was validated in other languages, delineating the need for further testing of the other instruments explored in this review.

The first instrument that measured the competencies of nurse managers was developed in 2006; most of them were developed during the last 17 years. This suggests that the study of competencies among NMs is still in its infancy and needs further exploration.

Scales developed more recently showed better quality in psychometric properties than previous ones due to the continuous updating of statistical techniques and the broader range of support literature compared to past years.

Management competencies are context-sensitive and influenced by the complexity of the sector, teams, and organisations in which these competencies must be demonstrated. Hence, most of the domains encountered in this review regarding this specific sector can also be commonly found in different nonhealthcare realities such as commercial banks [33], marketing [34], and the military [35]. As also demonstrated in the studies that consider a generic population of middle managers, not strictly related to the healthcare sector, communication, organisation, information searching, analytical thinking, and planning skills are typical yet required for good public middle managers. Achievement orientation, leadership, directiveness, persuasiveness, and creativity are qualities that separate good public middle managers from mediocre performance. Furthermore, some other new competencies obtained inductively through a thematic analysis are important for effective public managers: adherence to laws and regulations, multistakeholder collaboration, and technical competencies [36, 37].

Some domains overlapped across scales, while others with the same terminology included different skills. The number of domains (factors) varied between scales, from a minimum of 2 to a maximum of 11.

Learning from previous studies, the individual's educational and work background significantly impact developing competencies, representing a possible bias during the assessment [38]. However, having been a nurse manager does not adequately equip them for the vast range of abilities required, necessitating specialised training and practical work experience [39].

Regarding the COSMIN evaluation of the instrument quality, various critical aspects were identified that could potentially lead to ambiguous or unclear outcomes. For example, in 7 out of 10 studies, the sample size was below the recommended level based on the number of items included in the instrument. Also, insufficient attention was given to formulating an appropriate hypothesis regarding the expected correlations between competencies and the identified comparators, or the correlations needed proper confirmation [40, 41]. While exploratory factor analysis (EFA) was conducted to test scale dimensionality, confirmatory factor analysis (CFA) was performed in only a few studies before using the measurement instrument for research [42]. In two studies, Cronbach's alpha was calculated for the full scale instead of being calculated for each dimension in multidimensional instruments [43]. Addressing these methodological gaps is crucial for enhancing the validity and reliability of future studies employing these instruments. By summarizing the findings of this review, we can say that the study of competencies among NMs is still in its infancy and needs further exploration.

#### 3.4.1. Recommendation GRADE A

Despite criticism regarding weak structural validity, five instruments received a GRADE A recommendation. The studies of DeOnna [25], Tongmuangtunyatep et al. [28], Shuman et al. [29], Gunawan et al. [30], and Moghaddam et al. [27] exhibited satisfactory methodological quality in most of the measurement properties, and the quality of evidence ranged from low to high. In accordance with the COSMIN guidelines, these instruments are recommended for clinical practice use.

The NMCI questionnaire [25] received a positive assessment, but it exhibited deficiencies in some properties due to the lack of clarity in the stated method for data analysis. Nevertheless, it could serve as a foundation for sequencing competencies to develop an Middle Nurse Manager (MNM) orientation and career path program. The CASHN instrument scored highest in most of the measurement properties evaluated, with satisfactory methodological quality and high evidence level. Nurse executives can use this scale to plan the development of integrity and awareness of regulatory requirements for head nurses and to develop effective educational programs [28].

The Nurse Manager EBP Competency scale [29] can be used in complex and dynamic practice settings, explaining variations in implementing and sustaining EBP. An MNM's full competency in evidence-based management (EBM) may contribute to effectiveness in promoting good quality care. More research is needed on the reasons for and barriers to EBP implementation.

The I-FLNMMCS tool [30] can be used as a basis for FLNMs to improve their competence levels and as a vehicle for feedback mechanism; however, despite all psychometric properties being rated as sufficient and of moderate quality, structural validity was deemed insufficient due to an inadequate sample size.

Finally, the Questionnaire for Head Nurses' Managerial Competencies [27] was designed based on a proposed framework that included four main managerial tasks: planning, organising, leadership, and control. It showed limited evidence of structural validity: the sample size included in the analysis was inadequate [44]. Consistent with the findings of this study, a previous study conducted by Pillay [45] affirmed that head nurses should have strategic planning skills to set the vision, mission, goals, objectives, and strategies.

#### 3.4.2. Recommendation GRADE B

None of the included instruments received a GRADE C recommendation; however, five of them were categorised as GRADE B. The HCCI tool by Donaher et al. [24], the Home Healthcare Nurse Manager Assessment Tool [26], the Competency Elements for Nurse Managers of Tertiary General Hospitals by Wang et al. [22], the NICA-NL instrument developed by Yen et al. [42], and the Chase Nurse Manager Competency Instrument [5] received a GRADE B, primarily due to insufficient structural validity resulting from a small sample size. Notably, the NICA-NL instrument [42] distinguished itself as the sole tool specifically addressing informatics competencies. Validating these competencies equips nurse managers to personally contribute, rather than delegate, digital competencies to interprofessional initiatives, thereby fostering optimal and supportive care environments [23]. The Chase Nurse Manager Competency Instrument [5] holds the distinction of being the most widely used tool. The identified methodological gaps in these instruments underscore the necessity for additional testing to strengthen the validity and reliability of future studies employing these tools.

Thus, this systematic review of instruments measuring competencies among nurse managers confirms that management competencies can be broadly categorised as generic or specific to a particular profession [4]. Many competency domains encountered in the instruments included in the review are also commonly found in nonhealthcare sectors such as commercial banks [33], marketing [34], and the military [35], emphasizing the versatility of these competencies. Commonalities exist in competence domains such as communication, organisation, information searching, analytical thinking, and planning competencies [40]. Moreover, specific competencies such as adherence to laws and regulations, multistakeholder collaboration, and technical skills are important for managers in the healthcare sector [36, 37]. In addition, distinct levels of healthcare organisational management require varying competencies, including achievement orientation, leadership, directiveness, persuasiveness, and creativity [40]. These qualities are crucial for effective performance among top-middle managers in the healthcare sector, differentiating between good and mediocre performance in this context [40]. The interplay of educational and professional background further influences the development of competence and performance levels [38, 39]. Therefore, this comprehensive review lightens the multifaceted nature of management competencies among nurse managers, highlighting their relevance to the healthcare sector and underscoring the crucial need for specialised training in developing competencies for effective healthcare organisational management.

When designing new instruments, efforts should be made to standardize the instrument development process. Scale development and measurement property validation can be carried out by utilising standardized development techniques or the COSMIN guidelines. When developing scale items, the expert consultation approach should be supplemented with additional qualitative research methods, such as in-depth interviews and focus group discussions, to extensively investigate patients' viewpoints from different perspectives. Scale creation and measurement property evaluation should be guided by item response theory, classical measurement theory, and other relevant theories. In addition, a recall period should be established during scale creation to offer a useful reference for measuring scale responsiveness and to assure consistency and accuracy in the assessment.

### 3.5. Strengths and Limitations

The strength of this study lies in the methodological rigor applied to instrument evaluation and the resulting recommendation for the use of instruments with high quality. Researchers selected the COSMIN checklist, known for its rigorous assessment of methodological quality in reviewed studies, to fortify the methodology of this systematic review on measurement properties. Adhering to the PRISMA guidelines, two researchers independently ensured a standardized and coherent data selection process. The methodological quality of the included studies was individually assessed by reviewers, and a consensus was reached on the rating scores. A notable strength of this study lies in its objective to encompass available instruments measuring the competencies of nurse managers across various levels and the inclusion of diverse settings for evaluating the phenomena on a broad scale. All ten reviewed studies were conducted in heterogeneous public and private settings such as hospitals, primary care, home-based care, and acute and chronic care. This comprehensive approach spanned countries across four continents, enhancing the study's global relevance and applicability.

However, certain limitations need to be taken into account.

The search was limited to four databases, including English and Italian language studies. This may have excluded relevant studies written in other languages and indexed in other databases.

In addition, as measurement properties were extracted from published articles, the limited space available in journals could have restricted the reporting of instrument validation, affecting methodological quality assessment.

Since the COSMIN criteria for assessing the methodological quality of instrument measurement properties are highly detailed and rigorous, an instrument rated as poor or indeterminate could still be valid or reliable. The quality of reporting and the design of the validation studies should be improved by using, for example, COSMIN quality criteria as guidelines.

### 3.6. Implications and Recommendations

As already known, high-level nurse management competencies influence healthcare quality and relative outcomes. The results of this study could be significant for shaping the design of competency-based academic and training programs for nurse managers as well as for the development of competency assessment tools, performance appraisal tools, and staff recruitment strategies.

The results of this study may be used to design the professional roles of nurse managers, and to improve the leadership and management skills. Another remark is that nurse managers' research and development competencies still need to be improved since they have a crucial role in developing nursing care, despite the fact that their roles have evolved and become less clinical in some nations. Therefore, nursing personnel consider this role to be one of their most important contacts for communicating concerns and patient care requirements.

Although existing instruments may comprehend some of the fundamental domains, they do not set out to capture all elements of nurse management competence that may be important to consider. Indeed, a significant degree of heterogeneity was found in the definition of competency, and different synonyms were used for the domains studied and the competencies included in each of them, making it challenging to compare scales and their assessment methods. Therefore, future studies should try to unify competencies descriptions and interpretations to achieve consistency and a common language between countries.

We recommend advancing the study of the aforementioned instruments by contextualising them in other settings and countries. In addition, defining a cut-off point on the scales to assess the level of achievement and comparing it over repeated intervals can further enhance our understanding of specific knowledge and competence in nursing management.

## 4. Conclusions

This review aims to provide a meaningful understanding of existing instruments for measuring and evaluating the core competencies of first-, middle-, and top-level nurse managers.

Due to limited or unknown evidence about some measurement properties, the identified instruments should be used cautiously in clinical practice because of their variations.

Exactly half of the selected instruments were divided between GRADE A (Nurse Manager EBP Competency Scale, NMCI, CASHN, I-FLNMMCS, and Questionnaire for Head Nurses' Managerial Competencies) and B (HCCI, Chase Nurse Manager Competency Instrument, and Home Healthcare Nurse Manager Assessment Tool, NICA-NL, and Competency Elements for Nurse Managers of Tertiary General Hospitals) recommendations.

The difference between one level and the other lay in the accuracy and depth of the methodology chosen to validate the instrument, especially due to the inadequacy of the sample size.

Some competencies were commonly explored among instruments, including staff advocacy and development, team communication and cooperation, time management, quality improvement, leadership, problem resolution, and evidence-based practice. Other competencies, such as organisational and policy overview or negotiation, were less addressed.

The CASHN tool was the one with the highest score in both methodological quality and GRADE of evidence. The Chase Nurse Manager Competency Instrument was the one that included the most competencies.

Future research should focus on developing scales by using a more rigorous methodology, considering well-accepted theories to assess the different dimensions of management-related competencies and creating an inclusive definition for managerial competencies.

We also suggest completing the validation procedures started for both newly constructed and previously developed instruments but with higher-quality techniques and estimation of all psychometric features.

## Figures and Tables

**Table 1:** Literature search terms.

Database	Search terms	Number of articles (*n* = 789)
PubMed CINAHL	(“assess^*∗*^”[all fields] OR (“evalua^*∗*^”[all fields])) AND “competenc^*∗*^”[all fields] AND “nurse administrator”[MeSH terms] OR “nurse”[all fields] AND “nurse administrator^*∗*^”[all fields] OR (“nurs^*∗*^”[all fields] AND “manager”[all fields]) OR “nurse manage^*∗*^”[all fields] OR (“nurse lead^*∗*^”[Journal] AND (“coordinat^*∗*^”[all fields] OR (“nurse administrat^*∗*^”[MeSH terms] AND “executive^*∗*^”[all fields]) OR “nurse executive”[all fields])) AND “tool”[all fields] OR “instrument^*∗*^”[all fields] OR (“scale^*∗*^”[all fields] AND “measure^*∗*^”[all fields]) OR “inventor^*∗*^”[all fields] OR “questionnaire^*∗*^”[all fields] OR (“survey”[all fields])	351 249

Scopus	TITLE-ABS-KEY ((assessment OR evaluation OR monitoring) AND (competenc^*∗*^) AND (nurse AND manager OR nurse AND leader OR nurse AND coordinator OR nurse AND executive OR nurse AND administrator) AND (tool OR instrument OR scale OR inventory OR questionnaire))	81

APA PsycINFO	((any field: assessment OR any field: evaluation OR any field: monitoring) AND (any field: competenc^*∗*^) AND (any field: nurse AND any field: manager OR any field: nurse AND any field: leader OR any field: nurse AND any field: coordinator OR any field: nurse AND any field: executive OR any field: nurse AND any field: administrator) AND (any field: tool OR any field: instrument OR any field: scale OR any field: inventory OR any field: questionnaire))	108

**Table 2:** Characteristics and measurement properties of the competency assessment instruments.

Authors, year, country	Instrument/structure	Sample	Validity	Reliability
Wang et al., 2022 China [22]	Competency Elements for Nurse Managers of Tertiary General Hospitals-Chinese 22 items 5-point Likert scale 4 domains: Leadership and management ability, personal traits, professional quality, and professional ability	*n* = 518 Nurse managers: (i) Junior = 37 (ii) Middle = 316 (iii) Subsenior = 148 (iv) Senior = 17	*Content validity*: ECVI (expected cross-validity index) = 3.314 *Face* validity: pilot test on 136 subjects *Structural validity*: PCA with varimax rotation: 4 factors explaining 62.21% of the variance	*Internal consistency*: Cronbach's *α* = 0.745–0.885

Collins et al., 2017 USA [23]	NICA-NL-English 26 items 5-point Likert scale 6 domains: strategic implementation, advanced information and education, executive planning, ethical and legal concepts, information systems concepts, and requirements and system selection	*n* = 357 (i) Nurse manager = 74 (ii) Director = 103 (iii) Chief nursing officer = 38 (iv) Other = 142	*Content validity*: CVI = 0.96–1 *Face validity*: 3-round Delphi study on 101 subjects *Structural validity*: PAF with promax rotation: 6-factors	*Internal consistency*: Cronbach's *α* = 0.81–0.96

Chase, 2010 USA [5]	Chase Nurse Manager Competency Instrument-English 53 items 4-point Likert scale 5 domains: constructs, technical, human, conceptual, leadership, and financial management	*n* = 81 American Organization of Nurse Executives (AONE) members	*Content and face validity*: a panel of 53 experts *Structural validity*: PCA with varimax rotation: 5 factors explaining 57.2% of the variance	*Internal consistency*: Cronbach's *α* = 0.909 *Test*-*retest*: 2 weeks apart with 23 NMs (*r* = 0.88)

Donaher et al., 2007 USA [24]	HCCI-English 61 items 4-point Likert scale 5 domains: developing self, recruiting, developing others, utilising, and retaining	*n* = 99 (i) First-line = 53 (ii) Middle = 45	*Content validity*: CVI = 1.0 *Face validity*: a panel of 4 experts *Structural validity*: PCA with promax rotation: 3 factors explaining 68.87% of the variance	*Internal consistency*: Cronbach's *α* = 0.84–0.89

Gunawan et al., 2019 Indonesia [25]	I-FLNMMCS-Indonesian and English 43 items 5-point Likert scale 7 domains: leadership, facilitating spiritual nursing care, self-management, staffing and professional development, informatics, financial management, and applying quality care improvement	*n* = 300 first-line	*Content validity*: CVI = 0.859 *Face validity*: a panel of 7 experts and a pilot test on 17 subjects *Structural validity*: PCA with varimax rotation: 7 factors explaining 51.37% of the variance *Cross-cultural validity*: forward-backward translation method (CVI from 0.83 to 1) *Hypothesis testing*: (*r* = 0.321–0.687)	*Internal consistency*: Cronbach's *α* = 0.955

DeOnna, 2006 USA [26]	NMCI-English 93 items 5-point Likert scale 11 domains: promotes staff retention and recruitment, facilitates staff development, performs supervisory responsibilities, ensures patient safety and quality care, conducts daily unit operations, manages fiscal planning, facilitates interpersonal, group, and organisational communication, leads quality improvement initiatives, promotes professional practice model, and develops self	*n* = 527 (i) First-line = 309 (ii) Middle = 159 (iii) Executive = 48	*Content validity*: CVI = 1.0 *Face validity*: a panel of 7 experts *Structural validity*: PCA: 3 factors explained 68.87% of the variance	*Internal consistency*: Cronbach's *α* = 0.96

Moghaddam et al., 2019 Iran [27]	Questionnaire for Head Nurses' Managerial Competencies-Iranian 78 items 5-point Likert scale 4 domains: planning, organising, leadership, and control	*n* = 30 head nurses	*Content validity*: CVI = 0.7–0.79 *Face validity*: 2-round Delphi study on 16 experts and a pilot test on 30 subjects	*Internal consistency*: Cronbach's *α* = 0.93

Tongmuangtunyatep et al., 2015 Thailandia [28]	CASHN-Thailandese 52 items 5-point Likert scale 5 domains: leadership, healthcare environment management, policy implementation and communication, management, and professional ethics	*n* = 614 head nurses	*Content validity*: CVI = 0.94 *Face validity*: a panel of 6 experts and a pilot test on 30 subjects *Structural validity*: PCA with oblique rotation: 3 factors explained ≥60% of the variance	*Internal consistency*: Cronbach's *α* = 0.93–0.96

Shuman et al., 2017 USA [29]	Nurse Manager EBP Competency Scale-English 16 items 3-point Likert scale The total score is calculated by summing scores for all 16 items and then dividing by 16 2 domains: EBP knowledge and EBP activity	*n* = 83 nurse managers	*Face validity*: a panel of 8 experts and a pilot test on 4 subjects *Structural validity*: PAF with promax rotation: 2-factors	*Internal consistency*: Cronbach's *α* = 0.95

Rosenfeld et al., 2012 USA [30]	Home Healthcare Nurse Manager Assessment Tool-English 38 items 5-point BARS scale 5 domains: leadership, problem-solving, planning and organisation, coaching, and aligning performance for success	*n* = 57 supervisors (i) 31 directors (ii) 9 administrators (iii) 8 managers (iv) 9 others *n* = 154 (i) 123 manager (ii) 17 coordinators (iii) 10 specialists (iv) 4 others	*Face validity*: pilot test *Structural validity*: PCA with varimax rotation: 5 factors explained 66.9% of the variance	*Internal consistency*: Cronbach's *α* = 0.866–0.948

NICA-NL, Nursing Informatics Competency Assessment for the Nurse Leader; NMCCQ, Nursing Manager Communication Competency Questionnaire; NMLMC, Nurse Managers Leadership and Management Competencies Scale; HCCI, Human Capital Competencies Inventory; I-FLNMMCS, Indonesian First-Line Nurse Managers' Managerial Competence Scale; LaMI, Leadership and Management Inventory; NMCI, Chase Nurse Manager Competency Instrument; CASHN, Competency Assessment Scale for Head Nurses.

**Table 3:** The methodological quality of psychometric properties and level of evidence of included studies.

Authors (*no*. of reference)	Relevance	Comprehensiveness	Comprehensibility	Overall content validity	Structural validity	Internal consistency	Reliability	Construct validity	Recommendation
Wang et al., 2022 [22]	−/M	−/M	+/M	+/M	−/M	+/M	?/M		B
Collins et al., 2017 [23]	+/M	+/M	+/M	?/M	−/L	?/L			B
Chase, 2010 [5]	+/M	+/M	+/M	+/M	−/M	+/M	+/M	+/M	B
Donaher et al., 2007 [24]	+/L	−/L	?/L	−/L	−/L	+/L		+/L	B
Gunawan et al., 2019 [25]	+/M	+/M	+/M	+/M	−/M	+/M			A
DeOnna, 2006 [26]	+/M	+/M	+/M	+/M	−/L	+/L		+/L	A
Moghaddam et al., 2019 [27]	+/M	+/M	+/M	+/M		+/L	+/L		A
Tongmuangtunyatep et al., 2015 [28]	+/H	+/H	+/H	+/H	+/H	+/H		+/H	A
Shuman et al., 2017 [29]	+/M	+/M	+/M	+/M	?/M	+/M			A
Rosenfeld et al., 2012 [30]	−/M	+/M	+/M	+/M	−/VL	?/VL	+/VL		B

Methodological quality of the study measurement properties was rated as follows: + = sufficient; − = insufficient; and ? = indeterminate. Quality of evidence was rated as follows: H = high; M = moderate; L = low; and VL = very low.

**Table 4:** Domains associated with each instrument.

Categories	Wang et al., 2022	Yen et al., 2017	Chase, 2010	Donaher et al., 2007	Gunawan et al., 2019	DeOnna, 2006	Moghaddam et al., 2019	Tongmuangtunyatep et al., 2015	Shuman et al., 2017	Rosenfeld et al., 2012	*F*
Staff advocacy and development	•		•	•	•	•		•		•	7
Team communication and collaboration	•		•			•	•	•		•	6
Change and resource management		•	•				•	•	•		5
Quality care and patient safety	•		•		•	•		•			5
Personal mastery and self-development	•			•	•	•	•				5
Staff retention and recruitment			•	•		•			•		4
Performing supervisory responsibilities	•			•		•		•			4
Time management		•	•	•		•	•			•	6
Quality improvement			•		•	•	•	•	•		6
Promoting professional model			•			•	•				3
Group management			•	•			•	•			4
Achievement orientation	•		•	•			•			•	5
Organizational and political view			•								1
Leadership	•		•	•	•			•		•	6
Informatics		•	•		•						3
Financial management			•		•						2
Ethical and legal concepts		•	•		•			•			4
Negotiation							•				1
Evidence-based practice	•		•		•		•	•	•		6
Problem-solving			•		•		•	•	•	•	6
*F* ^*∗*^	8	4	17	8	10	9	11	11	5	6	

*F* ^*∗*^ = frequency of appearance.

## Data Availability

No data were used to support the study.

## Additional Points

*What is Already Known*? High-level nurse management competencies influence healthcare quality and help achieve organisational goals. Existing instruments do not include all elements of competence that may be important to measure. *What This Paper Adds*? The CASHN tool (Competency Assessment Scale for Head Nurses) is the most recommended and effective methodological instrument for evaluating the competencies of nurse managers. A more rigorous methodology should be employed to conduct psychometric validation for both newly constructed and previously developed nurse manager competence instruments.
